# Information extraction pipelines for knowledge graphs

**DOI:** 10.1007/s10115-022-01826-x

**Published:** 2023-01-07

**Authors:** Mohamad Yaser Jaradeh, Kuldeep Singh, Markus Stocker, Andreas Both, Sören Auer

**Affiliations:** 1 L3S Research Center, Leibniz University Hannover, Hanover, Germany; 2 Zerotha-Research and Cerence GmbH, Aachen, Germany; 3 TIB Leibniz Information Centre for Science and Technology, Hanover, Germany; 4 Anhalt University of Applied Sciences, Bernburg, Germany

**Keywords:** Information extraction, NLP pipelines, Software reusability, Semantic search, Semantic web

## Abstract

In the last decade, a large number of knowledge graph (KG) completion approaches were proposed. Albeit effective, these efforts are disjoint, and their collective strengths and weaknesses in effective KG completion have not been studied in the literature. We extend Plumber, a framework that brings together the research community’s disjoint efforts on KG completion. We include more components into the architecture of Plumber  to comprise 40 reusable components for various KG completion subtasks, such as coreference resolution, entity linking, and relation extraction. Using these components, Plumber dynamically generates suitable knowledge extraction pipelines and offers overall 432 distinct pipelines. We study the optimization problem of choosing optimal pipelines based on input sentences. To do so, we train a transformer-based classification model that extracts contextual embeddings from the input and finds an appropriate pipeline. We study the efficacy of Plumber for extracting the KG triples using standard datasets over three KGs: DBpedia, Wikidata, and Open Research Knowledge Graph. Our results demonstrate the effectiveness of Plumber in dynamically generating KG completion pipelines, outperforming all baselines agnostic of the underlying KG. Furthermore, we provide an analysis of collective failure cases, study the similarities and synergies among integrated components and discuss their limitations.

## Introduction

Since the early twenty-first century [8], there have been continuous efforts to extend the Web with a global data graph using the Resource Data Framework (RDF) to publish structured data on the Web. One of the pivotal steps in this effort was the emergence of publicly available Knowledge Graphs (KG) such as DBpedia [4] and Yago [28] as large sources of structured data. Since then, these KGs have become a rich source of structured content used in various applications, including Question Answering (QA), fact checking, and dialog systems [6]. The research community addresses the problem of populating a KG from multiple angles, one of them is the semantic labeling of (semi-)structured data [1, 60], others use unstructured text to populate a KG. We focus on the latter efforts in our work. Numerous approaches to extract triple statements [66], keywords/topics [16, 17], tables [36, 37, 67], or entities [54, 55] from unstructured text to complement KGs have been developed by the community. Despite extensive research, public KGs are not exhaustive and require continuous effort to align newly emerging unstructured information to the concepts of the KGs.

In this article, we extend our previous work in [40] in the following regards:

- We formalize the workflow of the framework and include details of how components are selected and how pipelines are generated.
- We further include eight new components to the list of community-created components that Plumber  can use to generate dynamic information extraction pipelines.
- We evaluate further on another widely used KG (Wikidata) with a big dataset that is used by the community.
- We further analyze error rates, time efficiency, and conduct a more detailed ablation study on the newly supported dataset and the old ones. Furthermore, we show how the framework can be used in a human-in-the-loop workflow to improve extractions and results.

### Research problem

This work was motivated by an observation with recent approaches [20, 26, 54, 55, 67] that automatically align unstructured text to structured data on the Web. Such approaches are not viable in practice for extracting and structuring information because they only address very specific subtasks of the overall KG completion problem. If we consider the exemplary sentence *Rembrandt painted The Storm on the Sea of Galilee. It was painted in 1633.* To extract statements aligned with the DBpedia KG from the given sentences, a system must first recognize the entities and relation surface forms in the first sentence. The second sentence requires an additional step of coreference resolution, where *It* must be mapped to the correct entity surface form (namely, *The Storm on the Sea of Galilee*). The last step requires mapping of entity and relation surface forms to the respective DBpedia entities and predicates.

There has been extensive research in aligning concepts in unstructured text to KG, including entity linking [26, 30, 35], relation linking [6, 55, 57], and triple classification [25]. However, these efforts are disjoint, and little has been done to align unstructured text to the complete KG triples [41, 64]. Furthermore, many entity and relation linking tools have been reused in pipelines of QA systems [42, 58]. The literature suggests that once different approaches put forward by the research community are combined, the resulting pipeline-oriented integrated systems can outperform monolithic end-to-end systems. For example, Liang et al. [43] propose a modular QA system built reusing a variety of existing NLP components that outperform all existing end-to-end methods on the DBpedia-based QA task. For the KG completion task, however, to the best of our knowledge, approaches aiming at dynamically integrating and orchestrating various existing components do not exist.

### Objective and contributions

**Fig. 1:**
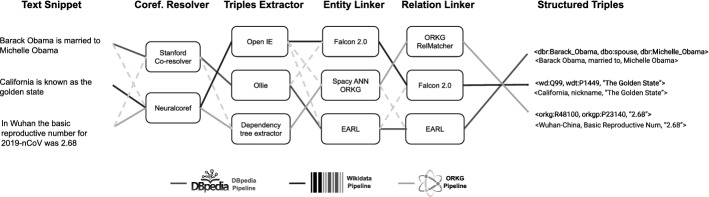
Plumber in action: three information extraction pipelines that convert natural language text into structured triples aligned with respective knowledge graphs. The optimal pipeline for each text snippet and corresponding KG is highlighted. Throughout the article, we use CR, coreference resolution; TE, triple extraction; EL, for entity linking; RL, relation linking

Based on these observations, we dynamically assemble and evaluate information extraction pipelines from previously disjoint efforts on the KG completion task under a single umbrella. We present the Plumber framework (originally described here [40], cf. Fig. 1) for creating Information Extraction (IE) pipelines for KG completion. Plumber integrates 40 reusable components released by the research community for the subtasks entity linking, relation linking, text triple extraction (subject, predicate, object), and coreference resolution. Overall, there are 432 different composable KG completion pipelines^1^ (generated by the possible combination of the available 40 components). Plumber implements a transformer-based classification algorithm that intelligently chooses the best pipeline based on the unstructured input text.

We perform an exhaustive evaluation of Plumber on the three large-scale KGs DBpedia, Wikidata [63] and Open Research Knowledge Graph (ORKG) [38] to investigate the efficacy of Plumber in creating KG triples from unstructured text. We demonstrate that independently of the underlying KG, Plumber can find and assemble different extraction components to produce optimized KG triple extraction pipelines, significantly outperforming existing baselines. In summary, we provide the following novel contributions:

- The Plumber framework is the first of its kind for dynamically assembling and evaluating information extraction pipelines based on sequence classification techniques and for a given input text. Plumber is easily extensible and configurable, thus enabling the rapid creation and adjustment of new information extraction components and pipelines. Researchers can also use the framework for running IE components independently for specific subtasks such as triple extraction and entity linking.
- A collection of 40 reusable IE components that can be combined to create 432 distinct IE pipelines.
- The exhaustive performance evaluation and our detailed ablation study of the integrated components and composed pipelines on various input text will guide future research for collaborative KG completion.

The article is organized as follows: Section 2 motivates our work. Related work is reviewed in Sect. 3, and we formalize our approach along with the problem definition in Sect. 4. Section 5 presents Plumber, which is evaluated in Sect. 6. Section 7 discusses the results. Section 8 concludes and outlines directions for future work.

## Motivating example

Let us consider as a running example the sentence *Rembrandt painted The Storm on the Sea of Galilee. It was painted in 1633*. The sentence can be represented using the DBpedia vocabulary as follows:
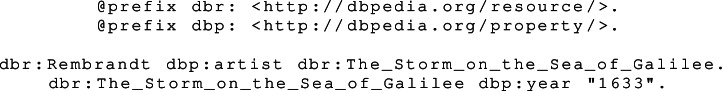


Multiple steps are required to extract these formally represented statements from the given text. First, the pronoun *it* in the second sentence should be replaced by *The Storm on the Sea of Galilee* using a coreference resolver. Next, a triple extractor should extract the correct text triples from the natural language text, i.e., <Rembrandt, painted, The Storm on the Sea of Galilee>, and <The Storm on the Sea of Galilee, painted in, 1633>. In the next step, the entity and relation linking component aligns the entity and relation surface forms extracted in the previous step to the DBpedia entities: dbr:Rembrandt for *Rembrandt van Rijn*, and dbr:The_Storm_on_the_Sea_of_Galilee for *The Storm on the Sea of Galilee*, and for relations: dbp:artist for *painted*, and dbp:year for *painted in*.

**Fig. 2:**
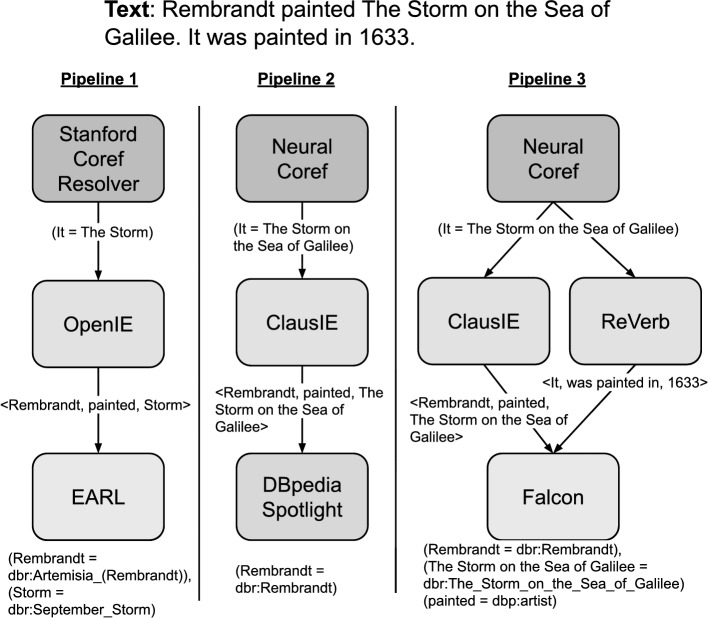
Three example information extraction pipelines showing different results for the same text snippet. Each pipeline consists of coreference resolution, triple extractors, and entity/relation linking components. For the sake of readability, we hide some intermediate triples and mappings. DBpedia was chosen over other KGs because it has human-readable URIs. The first layer from the top denotes the coreference resolution components in the pipelines. The middle one refers to textual triple extractors in the pipelines. And the bottom one indicates joint entity and relation linkers

There exists a plethora of techniques and components for extracting such statements from a given text. However, the performance of the tools varies widely and depends strongly on the input text (cf. [58]). Figure 2 illustrates our running example and shows three Plumber IE pipelines with different results. In Pipeline 1, the coreference resolver is unable to map the pronoun *it* to the respective entity in the previous sentence. Moreover, the triple extractor generates incomplete triples, which also hinders the task of the entity and relation linker in the last step. Pipeline 2 uses a different set of components, and its output differs from the first pipeline. Here, the coreference resolution component is able to correctly co-relate the pronoun *it* to *The Storm on the Sea of Galilee* and extract the text triple correctly. However, the overall result is only partially correct because the second triple is not extracted. Also, the entity linking component is not able to spot the second entity. It is important to note that the entity linking component in the second pipeline (i.e., DBpedia Spotlight [18]) does not perform relation linking. Hence, even if the information extraction step produces the correct results, triples could not be mapped correctly.

Pipeline 3 correctly extracts both triples. This pipeline employs the same component as the second pipeline for coreference resolution but also includes an additional information extraction component (i.e., ReVerb [29]) and a joint entity and relation linking component, namely Falcon [54]. With this combination of components, the text triple extractors were able to compensate for the loss of information in the second pipeline by adding one more component. Using the extracted text triples, the last component of the pipeline, a joint entity and relation linking tool, can map both triple components correctly to the corresponding KG entities.

With the availability of a large pool of components, such as those employed in Plumber, a suitable pipeline for a given text can be identified experimentally by executing all possible pipelines. However, this brute force approach is impractical. Therefore, we suggest a machine-learning model (cf. Section 4.3) for identifying a suitable candidate pipeline for a given input text.

## Related work

In the last decade, many open-source tools have been released by the research community to tackle IE tasks in the context of KG completion (see Table 2). These IE components are not only used for end-to-end KG triple extraction but also for various other tasks, such as:

### Text triple extraction

The task of open information extraction is a well-studied researched task in the NLP community [3]. It relies on NER (Named Entity Recognition) and RE (Relation Extraction). MinIE [34] extracts relation surface forms. SalIE [52] uses MinIE in combination with PageRank and clustering to find facts in the input text. Furthermore, OpenIE [3] leverages linguistic structures to extract self-contained clauses from the text. For a detailed survey on open information extraction, we point readers to a comprehensive survey by Niklaus et al. [51].

Another system Graphene [11] employs two layered transformations of clausal and phrasal embedding to simplify text and extract linguistic triples.

### Entity and relation linking

Entity and relation linking is a widely studied researched topic in the NLP, Web, and Information Retrieval research communities [5, 6, 20]. Often, entity and relation linking is performed independently. DBpedia Spotlight [18] is one of the first approaches for entity recognition and disambiguation over DBpedia. TagMe [30] links entities to DBpedia using in-link matching to disambiguate candidates entities. Open Tapioca [20] uses semantic matching of entity candidates for Wikidata. Other tools such as RelMatch [57] do not perform entity linking and only focus on linking the relation in the text to the corresponding KG relation. Recon [6] assumes entities are already linked in the text and aims to map relations between the entities to the KG using a graph neural network. EARL [26] is a joint linking tool over DBpedia and models the task as a generalized traveling salesperson problem. Sakor et al. [54] proposed Falcon, a linguistic rule-based tool for joint entity and relation linking over DBpedia. Falcon 2.0 [55] performs joint entity and relation linking on Wikidata.

### Coreference resolution

This task is used in conjunction with other tasks in NLP pipelines to disambiguate text and resolve syntactic complexities. The Stanford Coreference Resolver [53] uses a multipass sieve of deterministic coreference models. Clark and Manning [15] use reinforcement learning to fine-tune a neural mention-ranking model for coreference resolution. More recently, Sanh et al. [56] introduced a hierarchical model that is capable of multitask learning including coreference resolution.

### Frameworks and dynamic pipelines

There have been few attempts in various domains aiming to consolidate the disjoint efforts of the research community under a single umbrella for solving a particular task. The Gerbil platform [62] provides an easy-to-use web-based platform for the agile comparison of entity linking tools using multiple datasets and uniform measuring approaches. OKBQA [42] is a community effort for the development of multilingual open knowledge base and QA systems. Frankenstein integrates 24 QA components to build QA systems collaboratively on-top of the Qanary integration framework [10]. Plumber is closely inspired by the concept of Frankenstein but also differs in several ways. Firstly, Plumber surpasses Frankenstein in the number of integrated components. Furthermore, Frankenstein implements one classifier per component to predict the performance of each component for a given input question. Frankenstein treats each task independently. Hence, even if a classifier predicts that a specific component should be a part of the pipeline, it may be possible that the error propagated from previous steps reduces the overall performance. We argue that for selecting the best pipeline it is crucial to consider the end-to-end performance instead of treating each component independently. Hence, we trained a single transformer-based classifier that not only learns from the linguistic features but also from the results of other pipelines while suggesting the optimal pipeline. The results in Table 5 support our hypothesis, which is fundamentally different from the Frankenstein approach.

### End-to-end extraction systems

More recently, end-to-end systems are gaining more attention due to the boom of deep learning techniques. Such systems draw on the strengths of deep models and transformers [23, 44]. Kertkeidkachorn and Ichise [41] present an end-to-end system to extract triples and link them to DBpedia. Other attempts such as KG-Bert [66] leverage deep transformers (i.e., BERT [23]) for the triple classification task, given the entity and relation descriptions of a triple. KG-Bert does not attempt end-to-end alignment of KG triples from a given input text. Liu et al.  [45] design an encoder–decoder framework with an attention mechanism to extract and align triples to a KG.

## Approach formalization

An end-to-end information extraction pipeline is composed of all IE tasks (i.e., KG completion subtasks) needed to transform a sequence of natural language text into a set of structured triples in the form of (*subject, predicate, object*). However, since each component of the IE pipeline performs different tasks, we first formalize the interfaces of the IE tasks. We then state the problem, a formal approach implemented in Plumber  and how pipelines are generated.

### Defining various IE task interfaces

We formally define a pipeline *P* as a triple extraction and alignment function, from text *T* to a set of aligned KG triples $\Upsilon$.

1 $$\begin{aligned} P: T \rightarrow \Upsilon \end{aligned}$$

A text element *T* is a white-spaced separated sequence of words and sentences. Let $\oplus$ be the composition operator (i.e., the input of a function is the output of the previous one). We define *P* as a composition of four subfunctions, each corresponding to an IE task in the pipeline:

2 $$\begin{aligned} P:= \Theta \oplus Z \oplus \Psi \oplus \Omega \end{aligned}$$

An overview of the notation used in the problem formalization can be found in Table 1.

**Table 1:** Symbol notation used to formalize Plumber pipelines interfaces

*P*	IE extraction pipeline *P* composed of several IE components
$\Theta$, $\theta$	Set of coreference resolution (CR) components $\Theta$ and individual CR components $\theta \in \Theta$
*Z*, $\zeta$	Set of triple extractor (TE) components *Z* and individual TE components $\zeta \in Z$
$\Psi$, $\psi$	Set of entity linking/relation linking components $\Psi$ and individual EL/RL components $\psi$
$\Omega$, $\omega$	Set of end-to-end (E2E) components $\Omega$ and individual E2E components $\omega$
$\Upsilon$, $\upsilon$	Set of KG triples $\Upsilon$ and individual KG triple $\upsilon$
$\Lambda (.)$	Transformation function that enriches a text *T*
*c*	Coreference chain $c= \{m, a\}, m:= Mention, a:= Alias$
$\gamma$	Text triple $\gamma = <s, p, o>, s:= subject, p:= predicate, o:=object$
$\lambda$	Mapping pair $\lambda = (m, u), m:= Mention, a:= \text {KG }uri$
*K*	Knowledge Graph *K* also referred to as Knowledge Base

*(i) Coreference resolution (CR)* The first step is to disambiguate the input text and replace pronouns and acronyms with its associated entity mention. This step is formally defined as:

3 $$\begin{aligned} \theta := T \rightarrow T', T':= \Lambda (T,c), c:=\{(m, a) \subseteq T| m,a \ne \emptyset \} \end{aligned}$$

where $T'$ is a text resulting from the transformation function $\Lambda$ and the coreference chain *c*. *m* is the mention in the text *T* and *a* is the pronoun or other alias that refers to the mention *m*. The resulting text $T'$ is a text without ambiguities in mentions and pronouns.

*(ii) Text triple extraction (TE)* For the second step, we define a text triple as combination of three keyphrases or text snippets usually in the form of (subject, predicate, object). TE components extract such textual triples from the disambiguated text $T'$. Formally:

4 $$\begin{aligned} \zeta := T' \rightarrow {\bar{T}}, {\bar{T}}:= \Lambda (T',\gamma ), \gamma :=\{(s,p,o) \in T' | s,p,o \ne \emptyset \} \end{aligned}$$

Each triple set $\gamma$ is formed of triplets (i.e., {*s*, *p*, *o*}). The transformation function $\Lambda$ in this step enriches the disambiguated text $T'$ with set of triples producing ${\bar{T}}$.

*(iii) Entity and relation linking (EL/RL)* The third and concluding step in the pipeline is the alignment of triples in ${\bar{T}}$ to a knowledge graph *K*. We define a KG triple $\upsilon \in \Upsilon$ similarly to a text triple $\gamma$ whereby they have the same structure alike {*s*, *p*, *o*}. However, a KG triple assumes that the subject *s* and predicate *p* components are URIs referring to a KG *K*, and the object *o* is either a URI as well or a literal value (i.e., string snippet). Formally, it is defined as:

5 $$\begin{aligned} \begin{aligned} \psi&:= {\bar{T}} \rightarrow \Upsilon , \Upsilon := M({\bar{T}},\lambda , K), \\ \lambda&:=\{(m,u), m \in {\bar{T}}, u \subset K | m, u \ne \emptyset \} \end{aligned} \end{aligned}$$

Here $M({\bar{T}},\lambda , K)$ denotes a mapping function that takes the enriched text ${\bar{T}}$, a knowledge graph *K* (which is constant), and a set of mappings $\lambda$ to construct a set of aligned KG triples $\Upsilon$.

*(iv) End-to-end extraction (E2E)* resembles the composed pipelines *P* and is defined as $\omega := T \rightarrow \Upsilon$. End-to-End pipelines produce results that are concatenated to results of the $\Psi$ components.

### Generating candidate IE pipelines

Generating candidate pipelines relies on the interfaces of the IE tasks and the set of requirements *r*. The set of pipelines $\xi (r)$ is populated with candidate pipelines $\varrho _{i}$ following a composition:

6 $$\begin{aligned} \varrho _{i}:= \oplus _{\tau \in \Delta } \left\{ \chi _{r}^{\tau } \right\} \end{aligned}$$

where $\Delta$ the list of IE tasks following the specifications of the interfaces formalized previously. $\chi _{r}^{\tau }$ a set of possible IE components that perform the IE task $\tau$ and comply with the requirements *r* (i.e., the knowledge graph *K*). It is created by concatenating IE components carrying out a task $\tau$ (i.e., concatenating components is running them in parallel and concatenating the results). If $\mathbin \Vert$ denotes the concatenation of IE components, then the set of IE components $\chi$ is defined as follows:

7 $$\begin{aligned} \chi _{r}^{\tau }:= \mathbin \Vert _{i} \left\{ C(\tau ) \right\} , \text {for } i=1,\ldots , n \end{aligned}$$

where $C(\tau )$ retrieves a component from the set of IE components addressing task $\tau$ and *n* is the number of needed components per task. The *n* parameter is introduced to limit the space of candidates generation. Hence, pipelines can be generated and added to the pool of IE pipeline selection mechanism.

### Determining suitable IE pipeline

#### Problem

Here we tackle the pipeline selection problem. The pipeline selection problem deals with finding a suitable pipeline of IE components $\rho ^{r}_{s}$, for a sequence of text *s* and a set of requirements *r*. Formally, we define the optimization problem as follows:

8 $$\begin{aligned} \rho ^{r}_{s}:= \arg \max _{\varrho \in \xi (r)} \{{\mathcal {Q}}(\varrho , s)\} \end{aligned}$$

where $\xi (r)$ constitute the set of IE extraction pipelines that conform to the requirements *r* and ${\mathcal {Q}}(\varrho , s)$ corresponds to the estimated performance of a pipeline $\varrho$ on a text sequence *s*.

#### Solution

We understand the problem at hand as a *k*-class classification problem [46]. In order to be able to solve this problem, we decompose it into a series of smaller and simpler two-class problems. Suppose we have *k* pipelines (i.e., classes). Let ${\mathcal {W}}$ be the training set for a *k*-class problem:

9 $$\begin{aligned} {\mathcal {W}}:= \left\{ (X_{l}, Y_{l}) \right\} _{l=1}^{L} \end{aligned}$$

where $X_{l}$ is an input text sequence, $Y_{l} \in {\mathbb {R}}^{k}$ is the desired output, and *L* is the number of training data. Following the class decomposition methods [2, 14, 31], a *k*-class problem can be divided into *k* two-class problems. The training set for each of the two-class problems is as follows:

10 $$\begin{aligned} {\mathcal {W}}_{i}:= \left\{ (X_{l}, y_{l}^{i}) \right\} _{l=1}^{L}, \text {for }i=1,..., k \end{aligned}$$

where $y^{i}_{l} \in {\mathbb {R}}^{1}$ is the desired output defined as:

11 $$\begin{aligned} y_{l}^{i} = \left\{ \begin{matrix} 1-\epsilon &{}, X_{l} \in {\mathcal {C}}_{i} \\ \epsilon &{}, X_{l} \in \bar{{\mathcal {C}}}_{i} \end{matrix} \right. \end{aligned}$$

where $\epsilon$ is a small positive real number and $\bar{{\mathcal {C}}}_{i}$ denotes all classes except ${\mathcal {C}}_{i}$. A sequence classifier $\Gamma$ can now be trained on the decomposed training dataset ${\mathcal {W}}$ and is able to classify the performance of a pipeline ${\mathcal {Q}}(\varrho , s)$ into a class $\kappa \in k$ that maps to a pipeline configuration (i.e., a set of IE components). This is the best pipeline to run on the input sequence *s*. Hence, we rewrite Eq. 8 as follows:

12 $$\begin{aligned} \rho ^{r}_{s}:= \arg \max _{\kappa \in k} \{ \Gamma (s, \xi (r)) \} \end{aligned}$$

which stands for a problem of classifying a sequence of text *s* based on a set of candidate pipelines $\xi (r)$.

## Dynamic pipelining framework

Plumber orchestrates and evaluates IE components to select the most suitable pipeline configuration based on the input text for the KG completion task. We now detail its architecture.

**Fig. 3:**
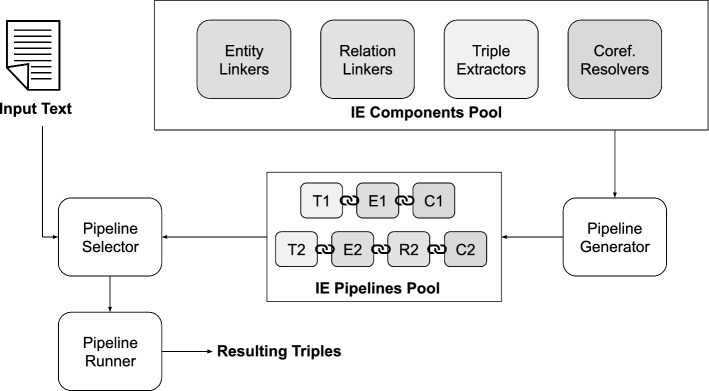
Overview of Plumber’s architecture highlighting the components for pipeline generation, selection, and execution. Plumber receives an input sentence and requirement (underlying KG) from the user. The framework intelligently selects the optimum pipeline based on the contextual features captured from the input sentence

### Architecture overview

Plumber has a modular design (see Fig. 3) where each component is integrated as a microservice. To ensure a consistent data exchange between components, the framework maps the output of each component to a homogeneous data representation using the Qanary [10] methodology. Qanary follows the linked data principles [7] employing an ontology to systematize the process of connecting components. Plumber follows three design principles: (i) *Isolation*, the IE components are independent from each other and they can be accessed through exchangeable interfaces; (ii) *Reusability*, the framework is open source and can be reused in different contexts and variations; (iii) *Extensibility*, Plumber provides common interfaces to expand and add further IE components in such a way that new components and tools are directly integrated in the framework and operate within the pipelines. The design of the framework is inspired by the work of [10, 58, 62]. The Qanary framework was used as basis for our architecture because it demonstrated why each of the previously mentioned principles is required, and it has been used extensively by other frameworks and systems such as [24, 58].

Plumber uses a RoBERTa [44]-based classifier that given a text and a set of requirements, it predicts the most suitable pipeline to extract KG triples (further details in Sect. 6.2).

Plumber includes the following modules:

- *IE components pool* All information extraction components that are integrated within the framework are parts of the pool. The components are divided based on their respective tasks, i.e., coreference resolution, text triple extraction, as well as entity and relation linking (cf. Table 2).
- *Pipeline generator* This module creates possible pipelines depending on the requirements of the components (i.e., the underlying KG). Users can manually select the underlying KG and, using the metadata associated with each component, Plumber aggregates the components for the concerned KG (cf. Sects. 4.1, 4.2).
- *IE pipelines pool* Plumber stores the configurations of the possible pipelines in the pool of pipelines for faster retrieval and easier interaction with other modules.
- *Pipeline selector* Based on the requirements (i.e., underlying KG) and the input text, a RoBERTa-based model extracts contextual embeddings from the text and classifies the input into one of the possible classes. Each class corresponds to one pipeline configuration that is held in the IE pipelines pool (cf. Sect. 4.3 for details of proposed optimization solution).
- *Pipeline runner* Given the input text, and the generated pipeline configuration, the module executes the pipeline to add the extracted triples to the KG.

Since Plumber  seeks to converge the disjoint efforts of the community under one umbrella, it does not reinvent the wheel or re-implement any IE component it encompasses. Plumber  relies on whatever interface the different IE components provide (see Table 2). In other words, if public APIs are available, then such interfaces are reused by the framework. However, if no API is available, then the IE component is integrated locally into the codebase (e.g., ClausIE & MinIE).^2^

## Evaluation

In this section, we detail the empirical evaluation of the framework in comparison with baselines on different datasets and knowledge graphs. As such, we study the following research question **RQ**: *How does the dynamic selection of pipelines based on the input text affect the end-to-end KG completion task?*. Throughout the paper, we use the term dynamic pipeline to refer to dynamic pipeline generation.

### Assumptions

Our empirical evaluation of the Plumber  framework is conducted with one assumption in mind. All externally accessible components (i.e., via APIs) are considered always operational, and any type of failure from their side is considered empty result set and treated accordingly in the evaluation procedure.

### Experimental setup

*Knowledge graphs* To study the effectiveness of Plumber in building dynamic KG completion pipelines, we use the following KGs during our evaluation:

*DBpedia* [4] (DBP) is containing information extracted automatically from Wikipedia info boxes. DBP consists of approximately 11.5B triples [54].

*Wikidata* [63] (WD) is a crowd-sourced knowledge base providing structured data for integration in Wikipedia. In contrast to DBP, WD also allows user-created entities and predicates. WD consists of over 4.9B triples [47].

*Open research knowledge graph* [38] (ORKG) collects structured scholarly knowledge published in research articles, using crowd sourcing and automated techniques. In total, ORKG consists of approximately 1.7 M triples.

*Datasets* Throughout our evaluation, we employed a set of both existing and newly created datasets for structured triple extraction and alignment to knowledge graphs: the WebNLG [33] dataset for DBP, the T-Rex [27] dataset for WD, and COV-triples for ORKG. The choice of datasets relied on three aspects: (i) the availability of the data (i.e., how much it is used in the community and the size of the data), (ii) the domain of the dataset (i.e., what is the dataset about), (iii) whether the selected IE components can handle the dataset (i.e., can extract entities and triples from it).

*WebNLG*^3^ is the Web Natural Language Generation Challenge. The challenge introduced the task of aligning unstructured text to DBpedia. In total, the dataset contains 46K triples with 9K triples in the testing and 37K in the training set.

*T-Rex*^4^ is a dataset of a large-scale alignment of Wikipedia text with Wikidata. It comprises approximately 11 M triples aligned to the WD knowledge graph. The data were split using an 80/20 ratio for training and testing.
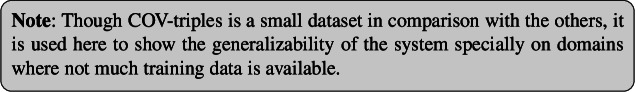


*COV-triples* is a handcrafted dataset that focuses on COVID-19-related scholarly articles. The COV-triples dataset consists of 21 abstracts from peer-reviewed articles and aligns the natural language text to the corresponding KG triples in the ORKG. Three semantic Web researchers verified annotation quality, and triples approved by all three researchers are part of the dataset. The dataset contains only 75 triples. Hence, we use the WebNLG dataset for training, and 75 triples are used as a test set.

*Components and implementation* The Plumber framework integrates 40 components, shown in Table 2 along with the associated subtasks and underlying KG.
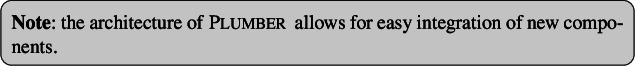


The IE components were chosen purely from the background knowledge of the authors and related work research. Our framework is implemented in Python, and we adapted a pre-trained version of RoBERTa from its public GitHub^5^ and fine-tuned it on the employed datasets. All experiments were performed on a system with 768GB RAM, 96 CPUs, and one GPU (NVIDIA GeForce 1080 Ti). The implementation code of Plumber and all related resources are publicly available online.^6^

**Table 2:** IE components implemented and integrated within the Plumber framework along with their respective publications, API links, underlying KGs, and respective task

Pipeline component	IE Task	Knowledge graph	Open source	Custom built	Rest API
*Ollie* [48]	TE	–	✓	✗	✗
*OpenIE * [3]	TE	–	✓	✗	✗
*ClausIE * [19]	TE	–	✓	✗	✗
*MinIE* [34]	TE	–	✓	✗	✗
*POS Extractor*$^\textrm{a}$	TE	–	✓	✓	✗
*Dependency Extractor*$^\textrm{b}$	TE	–	✓	✓	✗
*Graphene* [11]	TE	–	✓	✗	✗
*ReVerb *[29]	TE	–	✓	✗	✗
*R0 Extractor*	TE	ORKG	✓	✓	✗
*Stanford KBP Extractor* [12]	TE	–	✓	✗	✗
*Falcon *[54]	EL+RL	DBP	✓	✗	✓
*Falcon 2.0 * [55]	EL+RL	WD	✓	✗	✓
*EARL * [26]	EL+RL	DBP	✓	✗	✓
*Spacy ANN*$^\textrm{c}$	EL+RL	DBP+WD	✓	✓	✗
*Spacy ANN*$^\textrm{c}$	EL+RL	ORKG	✓	✓	✗
*Falcon NER * [54] + *ES*$^\textrm{d}$	EL+RL	DBP+WD	✓	✓	✗
*Falcon 2.0 NER + ES*$^\textrm{d}$	EL+RL	WD	✓	✓	✗
*EARL NER *[26] + *ES*$^\textrm{d}$	EL+RL	DBP+WD	✓	✓	✗
*Meaning Cloud*$^\textrm{e}$	EL	DBP	✗	✗	✓
*Text Razor*$^\textrm{f}$	EL	DBP+WD	✗	✗	✓
*DBpedia Spotlight *[18]	EL	DBP	✓	✗	✓
*TagMe *[30]	EL	DBP	✓	✗	✓
*OpenTapioca *[20]	EL	WD	✓	✗	✓
*TagMe NER *[30] + *ES*$^\textrm{d}$	EL	DBP+WD	✓	✓	✗
*Ambiverse-nlu *[35]	EL	WD	✓	✗	✗
*RelMatch *[57]	RL	DBP	✓	✗	✗
*Stanford Coref Resolver *[53]	CR	–	✓	✗	✗
*NeuralCoref *[15]	CR	–	✓	✗	✗
*PyCobalt*$^\textrm{g}$	CR	–	✓	✗	✗
*HMTL *[56]	CR	–	✓	✗	✗

$^\textrm{a}$Based on https://github.com/tdpetrou/RDF-Triple-API/

$^\textrm{b}$Adapted from https://github.com/anutammewar/extract_triplets/

$^\textrm{c}$https://github.com/microsoft/spacy-ann-linker/

$^\textrm{d}$https://www.elastic.co/elasticsearch/

$^\textrm{e}$https://www.meaningcloud.com/

$^\textrm{f}$https://www.textrazor.com/technology/

$^\textrm{g}$https://github.com/Lambda-3/PyCobalt

**Baselines** We include the following baselines:

- *T2KG* [41] is an end-to-end static system that aligns a given natural language text to DBpedia KG triples.
- *Frankenstein* [58] dynamically composes Question Answering pipelines over the DBpedia KG. It employs logistic regression-based classifiers for each component for predicting the accuracy and greedily composes a dynamic pipeline of the best components per task. We adapted Frankenstein for the KG completion over DBpedia and Wikidata since some of its components also perform entity and relation linking.
- *KnowledgeNet* [49] represents a benchmarking dataset for KG completion alongside a baseline model. The KnowledgeNet baseline model performs knowledge graph completion and population on the WD knowledge graph.

**Training the model** Plumber relies on a classification model to find a suitable pipeline. Each pipeline is represented as a class, making this a multiclass classification problem. To train the model, for every entry in the datasets, every possible pipeline is composed run on the input snippet.
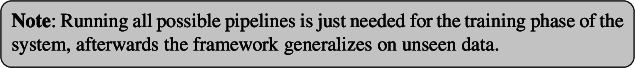


The results in terms of F1 scores are used to decide which pipeline performed better than the others. Next step for our underlying model (RoBERTa) is to create contextualized embeddings from the input text and learn to classify it into its corresponding class (i.e., the better performing pipeline from the IE pipelines pool). We choose a transformer-based architecture due to its ability to encode the contextual knowledge from the input text, providing a more accurate classification.

With the underlying model trained on the data, it can now dynamically compose information extraction pipelines on unseen data. However, if new IE components are added to the pool of available components, the framework would require the retraining of the model to include these new components as candidates.

### Experiments

This section summarizes a variety of experiments to compare the Plumber framework against other baselines. Note that evaluating the performance of individual components or their combination is out of this evaluation’s scope, since they were already used, benchmarked, and evaluated in the respective publications. We report values of the standard metrics precision (P), recall (R), and F1 score (F1) adapted from [13]. In all experiments, end-to-end components (e.g., T2KG) are not part of Plumber.

*Performance of static pipelines* In this experiment, we report results of the static pipelines, i.e., no dynamic selection of a pipeline-based on the input text is considered.
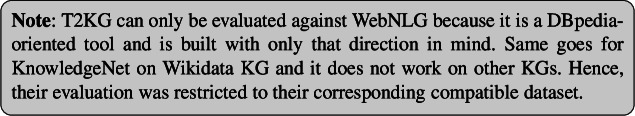


We ran all 432 pipelines, and Table 3 reports the performance of the best Plumber pipeline against the baselines. Plumber static pipeline for DBpedia comprises NeuralCoref [15] for coreference resolution, OpenIE [3] for text triple extraction, TagMe [30] for EL, and Falcon [54] for RL tasks. For Wikidata, the static pipeline contains NeuralCoref [15] for coreference resolution, Graphene [11] for text triple extraction, Falcon 2.0 [55] jointly for EL and RL tasks. Also, in case of Frankenstein, we choose its best-performing static pipeline. Results illustrated in Table 3 confirm that the static pipeline composed by the components integrated in Plumber outperforms all baselines on DBpedia and Wikidata. We observe that the performance of pipeline approaches is better than an end-to-end monolithic KG completion approaches. Although the Plumber pipeline outperforms the baselines, the overall performance is relatively low. All our components have been trained on distinct corpora in their respective publications, and our aim was to put them together to understand their collective strengths and weaknesses. Note Frankenstein addresses the QA pipeline problem and not all components are comparable and can be applied in the context of information extraction. Thus, we integrated the NeuralCoref coreference resolution component and the OpenIE triple extraction component used in the Plumber  static pipeline into Frankenstein in order to provide the same experimental settings.

**Table 3:** Performance comparison of the Plumber static pipeline against the baselines on different KGs

System	Dataset	Knowledge graph	P	R	F1	# Mapped triples
T2KG [41]	WebNLG	DBP	0.133	0.140	0.135	1.26K/9.0K
KnowledgeNet [49]	T-Rex	WD	0.243	0.254	0.247	0.56 M/2.2 M
Frankenstein [58]	WebNLG	DBP	0.177	0.189	0.181	1.70K/9.0K
	T-Rex	WD	0.228	0.249	0.238	0.55 M/2.2 M
Plumber	WebNLG	DBP	**0**.**210**	**0**.**225**	**0**.**215**	2.02K/9.0K
	T-Rex	WD	**0**.**282**	**0**.**296**	**0**.**289**	0.65 M/2.2 M

The total number of mapped triples in test set (Extracted/Expected) is given in the last column to indicate how many triples the systems produce regardless of correctness

*Static pipeline for scholarly KG* In order to assess how Plumber performs on domain-specific use cases, we evaluate the static pipelines’ performance on a scholarly knowledge graph. We use the COV-triples dataset for ORKG. To the best of our knowledge, no baseline exists on completing KGs of research contribution descriptions over ORKG. Hence, we execute all static pipelines in Plumber tailored to ORKG to select the best one as shown in Table 4. The best-performing pipeline over the COV-triples was composed of the HMTL [56] coreference resolution component in combination with our own custom created R0 Extractor and Spacy ANN joint entity and relation linkers.^7^Plumber pipelines over ORKG extract statements determining the reproductive number estimates for the COVID-19 infectious disease from scientific articles as shown below.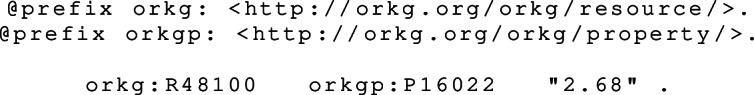 In this example, *orkg:R48100* refers to the city of Wuhan in China in the ORKG and *orkgp:P16022* is the property *has R0 estimate (average)*. The number “2.68” is the reproductive number estimate.

**Table 4:** Performance of the best-performing pipeline for scholarly knowledge extraction from COVID-19 research papers

System	Dataset	Knowledge graph	P	R	F1	# Mapped triples
Plumber	COV-triples	ORKG	0.403	0.423	0.413	32/75

*Comparison of the classification approaches for dynamic pipeline selection* In this experiment, we study the effect of the transformer-based pipeline selection approach implemented in Plumber against the pipeline selection approach of Frankenstein. For a comparable experimental setting, we re-use Frankenstein’s classification approach in Plumber, keeping the underlying components precisely the same. We perform a tenfold cross-validation for the classification performance of the employed approach. Table 5 demonstrates that the Plumber pipeline selection outperforms Frankenstein across all KGs.

**Table 5:** Tenfold cross-validation of pipeline selection classifiers wrt Precision, recall, and F1 score

Pipeline selection approach	Dataset	Knowledge graph	Classification		
			P	R	F1
Random	WebNLG	DBP	0.081	0.092	0.086
	T-Rex	WD	0.090	0.103	0.096
	COV-triples	ORKG	0.092	0.114	0.102
Frankenstein	WebNLG	DBP	0.732	0.751	0.741
	T-Rex	WD	0.770	0.791	0.780
	COV-triples	ORKG	0.832	0.858	0.845
Plumber	WebNLG	DBP	**0**.**877**	**0**.**900**	**0**.**888**
	T-Rex	WD	**0**.**891**	**0**.**912**	**0**.**901**
	COV-triples	ORKG	**0**.**901**	**0**.**917**	**0**.**909**

*Performance comparison for KG completion task* Our third experiment focuses on comparing the performance of Plumber against previous baselines for an end-to-end KG completion task. We also report the values of best-performing static pipelines from Table 3. The results in Table 6 illustrate that the dynamic pipelines built using Plumber for KG completion outperform the best static pipelines of Plumber as well as the dynamically selected pipelines by Frankenstein. The end-to-end baselines, such as [41, 49], significantly underperform compared to dynamic pipelines. We also observe that in cross-domain experiments for COV-triples datasets, dynamically selected pipelines perform better than the static pipeline. In the cross-domain experiment, the static and dynamic Plumber pipelines are relatively better-performing than the other two KGs. Unlike components for DBpedia and Wikidata, components integrated into Plumber for ORKG are customized for KG triple extraction. We conclude that when components are integrated into a framework such as Plumber aiming for the KG completion task, it is crucial to select the pipeline based on the input text dynamically. The superior performance of Plumber shows that the dynamic pipeline selection for KG completion has a positive impact agnostic of the underlying KG and dataset. This also answers our overall research question *How does the dynamic selection of pipelines based on the input text affect the end-to-end KG completion task?*.

**Table 6:** Overall performance comparison of static and dynamic pipelines for the KG completion task

System	Dataset	Knowledge graph	Performance		
			P	R	F1
T2KG [41]	WebNLG	DBP	0.133	0.140	0.135
KnowledgeNet [49]	T-Rex	WD	0.243	0.254	0.247
Frankenstein (static) [58]	WebNLG	DBP	0.177	0.189	0.181
	T-Rex	WD	0.228	0.249	0.238
Plumber (static)	WebNLG	DBP	0.210	0.225	0.215
	T-Rex	WD	0.282	0.296	0.289
	COV-triples	ORKG	0.403	0.423	0.413
Frankenstein (dynamic) [58]	WebNLG	DBP	0.199	0.208	0.203
	T-Rex	WD	0.244	0.263	0.253
	COV-triples	ORKG	0.403	0.424	0.413
Plumber (dynamic)	WebNLG	DBP	**0**.**287**	**0**.**307**	**0**.**297**
	T-Rex	WD	**0**.**361**	**0**.**397**	**0**.**378**
	COV-triples	ORKG	**0**.**411**	**0**.**437**	**0**.**424**

### Ablation studies

The performance of Plumber and baselines on all the employed datasets is relatively low. Hence, in the ablation studies our aim is to provide a holistic picture of underlying errors, collective success, and failures of the integrated components.

**Fig. 4:**
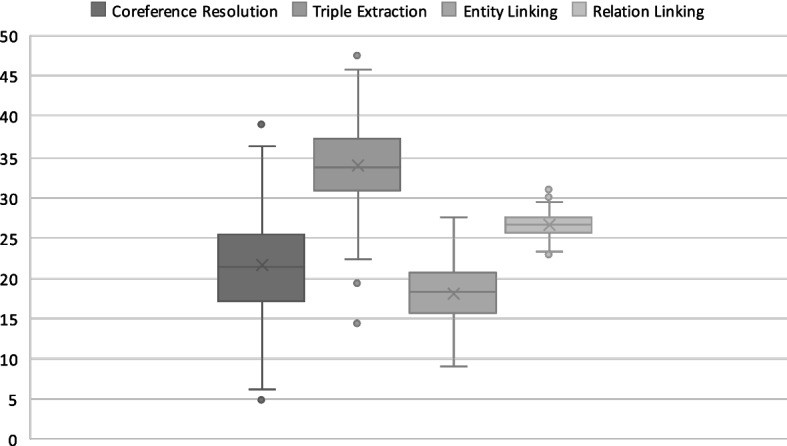
Box plot of error percentage per IE task. The Y axis shows the error percentage. Each box shows the error percentage by all components, the average error, and some of the outliers. Higher values mean a greater error rate. The figure shows that text triple extraction is the highest impacting component followed by relation linking, coreference resolution, and the least impacting is the entity linking

In the first study, we calculate the proportion of errors in Plumber. The modular architecture of the proposed framework allows us to benchmark each component independently. We consider the erroneous cases of Plumber on the test set of the WebNLG dataset. We calculate the performance (F1 score) of the Plumber dynamic pipeline (cf. Table 6) at each step in the pipeline. Figure 4 presents the results of the error evaluation. Each box in the figure corresponds to an IE task. The results show that the coreference resolution components caused 21.54% of the errors, 33.71% are caused by text triple extractors, 18.17% by the entity linking components, and 26.58% are caused by the relation linking components.

We conclude that the text triple extractor components contribute to the largest chunk of the errors over DBpedia. One possible reason for their limited performance is that open-domain information extracting components were not initially released for the KG completion task. Also, these components do not incorporate any schema or prior knowledge to guide the extraction. We observe that the errors mainly occur when the sentence is complex (with more than one entity and relation), or relations are not explicitly mentioned in the sentence. We further analyze the text triple extractor errors. The error analysis at the level of the triple subject, predicate, and object showed that most errors are in predicates (40.17%) followed by objects (35.98%) and subjects (23.85%).

*Further analysis* Aiming to understand why IE pipelines perform with low accuracy, we conduct a more in-depth analysis per IE task. In the first analysis, we evaluated each component independently on the WebNLG dataset. Researchers [21, 59] proposed several criteria for micro-benchmarking tools/components for KG tasks (entity linking, relation linking, etc.) based on the linguistic features of a sentence. We motivate our analysis based on the following criteria per task:

*Text triple extraction* We consider the number of words (*wc*) in the input sentence (a sentence is termed “simple” if it has an average word length of 7.41 [58]. Sentences with higher numbers of words than seven are complex sentences). Furthermore, having a comma in a sentence (subclause) to separate clauses is another factor. Atomic sentences (e.g., *cats have tails*) are a type of sentence that also affects triples extractors’ behavior. Moreover, nominal relation as in *Durin, son of Thorin* is another impacting factor on the performance. Uppercase and lowercase mentions of the words (i.e., correct capitalization of the first character and not the entire word) in a sentence are standard errors for entity linking components. We consider this a micro-benchmarking criterion.

*Coreference resolution* We focus on the length of the coreference chain (i.e., the number of aliases for a single mention). Additionally, the number of clusters is another criterion in the analysis. A cluster refers to the groups of mentions that require disambiguation (e.g., *mother bought a new phone, she is so happy about it* where the first cluster is *mother* $\rightarrow$ *she* and the second is *phone* $\rightarrow$ *it*). The presence of proper nouns in the sentence is studied as well as acronyms. Furthermore, the demonstrative nature of the sentence is also observed as a factor. Demonstrative sentences are the ones that contain demonstrative pronouns (this, that, etc.).

*Entity linking* The number (*e*= 1,2) of entities in a sentence is a crucial observation for the entity linking task. Capitalization of the surface form is another criterion for micro-benchmarking entity linking tools. An entity is termed as an explicit entity when the entity’s surface form in a sentence matches the KG label. An entity is implicit when there is a vocabulary mismatch. For example, in the sentence *The wife of Obama is Michelle Obama.*, the surface form *Obama* is expected to be linked to dbr:Barack_Obama and considered as an implicit entity [59]. The last linguistic feature is the number of words (*w*) in an entity label (e.g., *The Storm on the Sea of Galilee* has seven words).

*Relation linking* Similar to the entity linking criteria, we focus on the number of relations in a sentence (*rel*= 1,2).^8^ The type of relation (i.e., explicit or implicit) is another parameter. Covered relation (sentences without a predicate surface form) is also used as a feature for micro-benchmarking: *Which companies have launched a rocket from Cape Canaveral Air Force station?* where the dbo:manufacturing relation is not mentioned in the sentence. Covered relations highly depend on common sense knowledge (i.e., reasoning) and the structure of the KG [59]. Lastly, the number of words (*w*$>=\,$N) in a predicate surface form is also considered.

**Fig. 5:**
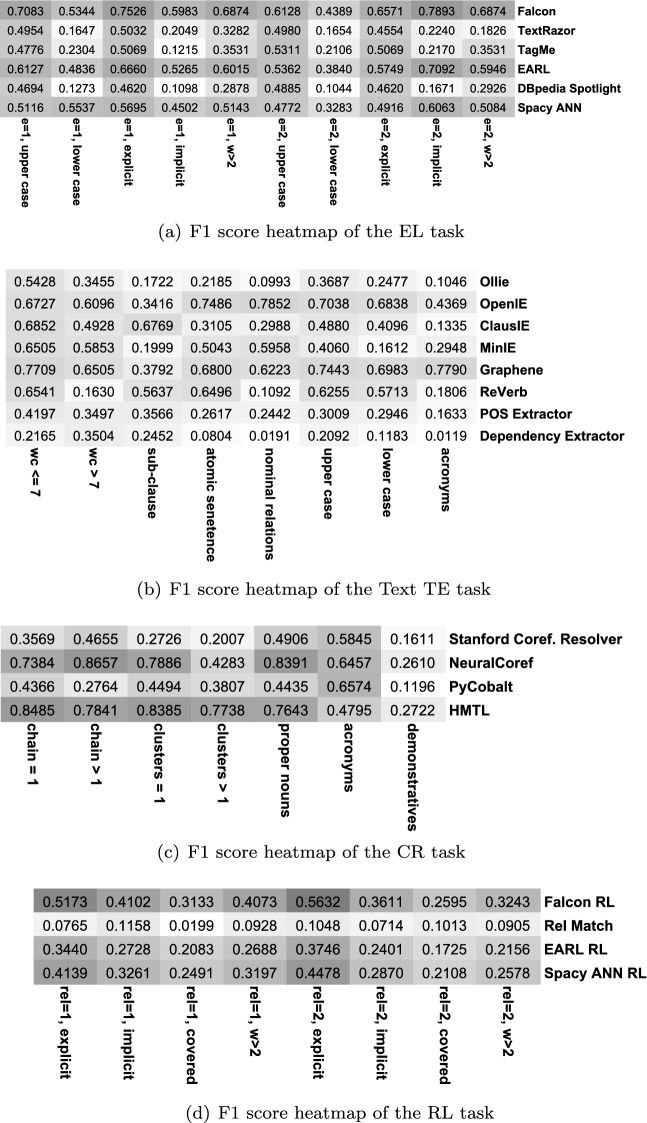
Comparison of F1 scores per component for different IE tasks based on the various linguistic features of an input sentence (number of entities, word count in a sentence, implicit vs. explicit relation, etc.). Darker shades indicate a higher F1 score

Figure 5 illustrates micro-benchmarking of various Plumber components per task. We observe that across IE tasks, the F1 score of the components varies significantly based on the sentence’s linguistic features. In fact, there exists no single component which performs equally well on all the micro-benchmarking criteria. This observation further validates our hypothesis to design Plumber for building dynamic KG completion pipelines based on the strengths and weaknesses of the integrated components.

In Fig. 5, all the CR components report limited performance for the demonstrative sentences (*demonstratives*). When there is more than one coreference cluster in a sentence, all other CR components observe a discernible drop in F1 score. The NeuralCoref [15] component performs best for *proper nouns*, whereas PyCobalt [32] performs best for the *acronyms* feature (almost being tied with NeuralCoref). In the TE task, Graphene [11] shows the most stable performance across all categories. However, the performance of all components (except Dependency Parser) drops significantly when the number of words in a sentence exceeds seven (*wc*>7). Case-sensitivity also affects the performance and all components observe a noticeable drop in F1 score for lowercase entity mentions in the sentence. A similar behavior is observed for entity linking components where case-sensitivity is a significant cause of poor performance. When the sentence has one entity and it is implicit (*e*= 1, *implicit*); all entity linking components face challenges in correctly linking the entities to the underlying KG. Relation linking components also report lower performance for implicit relations.

**Fig. 6:**
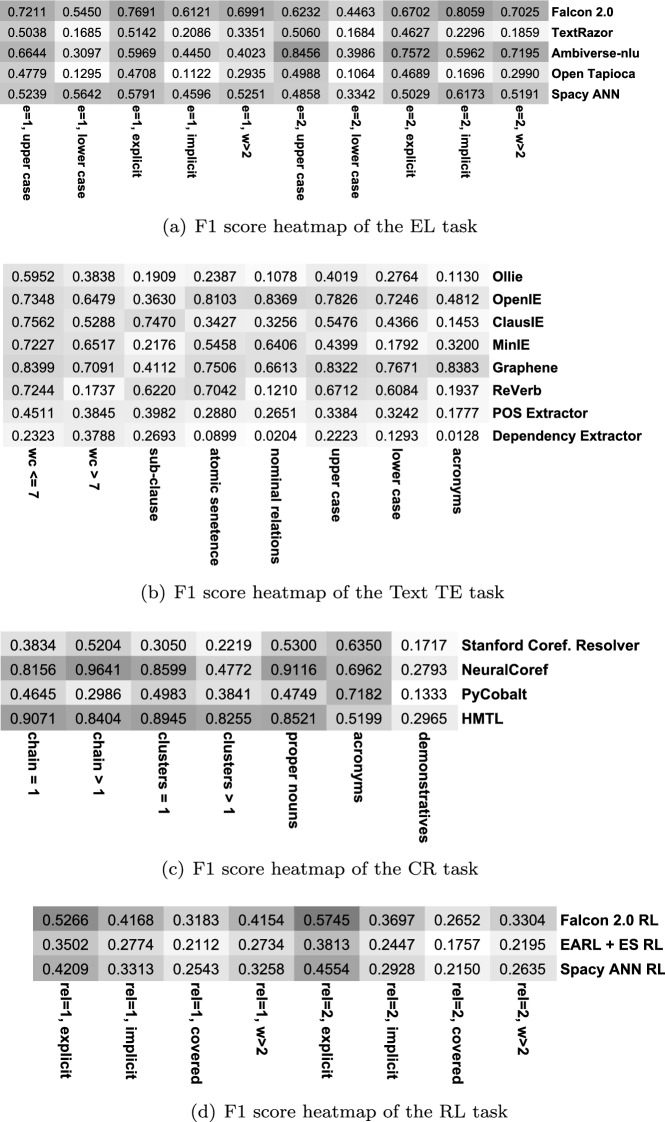
Comparison of F1 scores per component for different IE tasks (on the T-Rex dataset). Darker shades indicate a higher F1 score. We observe that components for Wikidata show a similar trend in the performance as in Fig. 5 where test sentences having linguistic features such as implicit entities, word count in a sentence, capitalization of entity surface form, etc. negatively impact performance

We then extended micro-benchmarking of the components to Wikidata and reported their performance in isolation. We considered all the sentences present in the T-Rex test set (approx 1.2 M sentences). Figure 6 illustrates the findings per linguistic feature for all IE subtasks. Similarly as for DBpedia, we observe that no single component is superior to all micro-benchmarking criteria. Issues such as capitalization of entity surface forms continue to impact EL and TE components’ overall performance negatively. Relation linking components on Wikidata inherit a similar trend as DBpedia components, where the implicit and hidden nature of relation surface forms has the highest impact on their performance.

**Table 7:** Average runtime on all datasets in seconds

System	WebNLG	T-Rex	COV-triples
T2KG [41]	2.8	–	–
KnowledgeNet [49]	–	3.4	–
Frankenstein (static) [58]	2.4	2.5	–
Frankenstein (dynamic) [58]	10.1	3.9	2.9
Plumber (static)	1.8	1.9	1.2
Plumber (dynamic)	12.3	3.9	2.7

The dynamic pipelines on WebNLG report the slowest runtime because few components have a high avg. runtime (up to 65 sec)

## Discussion

Even though the dynamic pipelines of Plumber outperform static pipelines, the overall performance of Plumber and baselines for the KG completion task remains low. Our detailed and exhaustive ablation studies suggest that when individual components are plugged together, their individual performance is a major error source. However, this behavior is expected, considering that earlier research works in other domains observe a similar trend. For example, since its first release in 2015, the research community performed over 50,000 experiments^9^ to improve EL components using the Gerbil framework [62]. Similarly, in 2018 Frankenstein reported the best dynamic question answering pipeline with F1 score 0.20. Within 2 years, the Semantic Web research community had released several components dedicated to solving entity linking and relation linking [26, 50, 54], which were two weaknesses identified by [58] for the QA task. At present, the QA system [43] reuses components from Frankenstein and is a new state of the art on standard complex QA dataset [61] with an F1 score of 0.68. We also calculated the average runtime of Plumber and baselines on all three datasets (cf. Table 7). The Plumber static pipeline was the fastest; however, the dynamic pipelines on DBpedia were the slowest. The main reason for the slow dynamic pipeline was the high runtime of DBpedia-based relation linking components. For example, Relmatch [57] has an average runtime of 65 s, thus negatively impacting the overall dynamic pipeline runtime. Due to the direct impact of a component’s runtime on the overall efficiency (runtime and memory consumption), improving the runtime efficiency of the Plumber pipelines was out of scope for this work. However, including Table 7 provides insight into how different baselines and systems perform with various datasets and data domains.

We observe that state-of-the-art components for KG completion still have much potential to improve their performance (both in terms of runtime and F1 score). It is essential to highlight that some of the issues observed in our ablation study basic and have been repeatedly pointed out by researchers in the community. For instance, Derczynski et al. [21] in 2015, followed by Singh et al. [58] in 2018, showed that case-sensitivity is a main challenge for EL tools. Our observations in Figs. 5 and 6 again confirm that case-sensitivity of entity surface forms remains an open issue even for newly released components. In contrast, on specific datasets such as CoNLL-AIDA, several EL approaches reported F1 scores higher than 0.90 [65], showing that EL tools are highly customized to particular datasets. In a real-world scenario like ours, the underlying limitations of approaches are uncovered. We also found that relation linking and text triple extractor components contributed caused significant errors in Plumber performance. Based on our findings, we identified the following open challenges for KG completion tasks, which we deem crucial to guide future research:

- The text triple extractor quality is low across KGs. We need to incorporate the KG’s underlying schema to guide the triple extraction.
- Case-sensitivity needs to be improved in entity linking approaches. Implicit entities also challenge the entity linking performance. Yang et al. [65] introduced entity descriptions as additional context to support implicit entity linking, and we deem such approaches to be beneficial for entity linking tools dedicated for an end-to-end KG completion.
- Relation linking accuracy is limited for the KG completion task across all micro-benchmarking features (cf. Figs. 5, 6). Handling implicit relations and covered relations are the primary source of errors, and we expect that our findings will motivate research to build dedicated relation linking components for KG completion.
- Overall, improving the component’s accuracy is the first viable next step for collaborative KG completion.

Furthermore, from a human in the loop approach, we envision employing this framework in conjunction with other knowledge management systems to allow users the possibility to leverage automated extraction from natural language text. With the possibility for user feedback on structured triple level, this information can be used in an active-learning style to improve the underlying pipeline selection model or even propagate these comments and feedback into the individual IE components for further improvement. In this use case, we integrate Plumber  within the ORKG infrastructure^10^ providing an access point to researchers to convert textual descriptions into structured and linked triples.

**Fig. 7:**
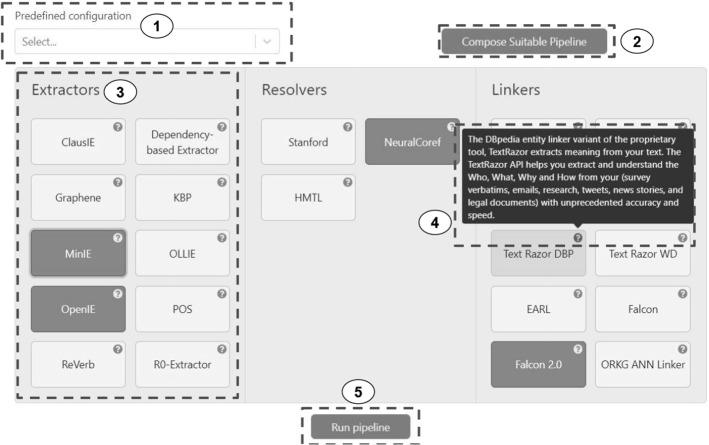
Overview of the user interface of Plumber in the ORKG infrastructure: (1) Predefined pipeline selector: used for easy access to generally stable information extraction pipeline, (2) invoke the framework to create a dynamic pipeline on-the-fly based on the input, (3) collection of IE components that can be used in conjunction manually or automatically, (4) additional information from components to better help the user interact with the system, (5) pipeline runner to display the results and get feedback

Figure 7 depicts how the Plumber can be integrated within other systems (here the ORKG). Moreover, such an integration allows for user feedback and comments to be fed into the system [39].

## Conclusion and future work

In this paper, we presented the Plumber approach and framework for KG completion. Plumber effectively selects the best possible pipeline for a given input sentence using the sentential contextual features and a state-of-the-art transformer-based classification model. Plumber has a service-oriented architecture which is scalable, extensible, reusable, and agnostic of the underlying KG. The core idea of Plumber is to combine the strengths of already existing disjoint research for KG completion and build a foundation for a platform to promote reusability for the construction of large-scale and semantically structured KGs. Our empirical results suggest that the performance of the individual components directly impacts the end-to-end KG completion accuracy.

This article does not focus on internal system architecture or employed algorithms in a particular IE component to analyze the failures. The focus of the ablation studies is to holistically study the collective success and failure cases for the various tasks. Our studies provide the research community with insightful results over three knowledge graphs, 40 components, 432 pipelines, and test datasets collectively containing over 2.2 M triples extracted from approximately 1.2 M sentences. We release all the experiment results publicly for reproducibility and continued research. Our work is a step in the larger research agenda of offering the research community an effective way for effective to synergistically combine and orchestrate various focused IE approaches balancing their strengths and weaknesses taking different application domains into account, applying their research to a domain driven by many different fields, consequently requiring a collaborative approach to achieve significant progress. We plan to extend our work in the following directions: (1) extending Plumber to other KGs such as UMLS [9] and AI-KG [22]. AI-KG also employs a single pipeline comprising community-released components for KG completion on their proposed scholarly KG. (2) adding multilingual components to Plumber, considering that existing components focus primarily on English, and (3) creating high performing relation linking components for KG completion.

## Footnotes

1 For DBpedia: 3 CRs, 8 TEs, and 10 EL/RLs, hence, $3*8*10=240$. And the same for Wikidata: 3CRs*8TEs*7EL/RL = 168. For the ORKG: 4CRs*3TEs*2EL/RL = 24 pipeline. In total: $240+168+24=432$.

2 See below for more details about such tools.

3 https://webnlg-challenge.loria.fr/.

4 https://hadyelsahar.github.io/t-rex/.

5 https://github.com/pytorch/fairseq/.

6 https://github.com/YaserJaradeh/ThePlumber.

7 Adaption of https://github.com/microsoft/spacy-ann-linker/, trained on the ORKG entites and relations.

8 This number of relations in inconsequential for a triple extractor, but it affects relation linkers and their complexity.

9 http://aksw.org/Projects/GERBIL.html.

10 Demo video: http://www.youtube.com/watch?v=XC9rJNIUv8g.
